# Learning-aided observer design for improving autonomous vehicle safety

**DOI:** 10.1038/s41598-026-35378-9

**Published:** 2026-03-03

**Authors:** András Mihály, Balázs Németh, Mihály Kopasz, Péter Gáspár, Ferenc Szauter

**Affiliations:** 1 Systems and Control Laboratory, Institute for Computer Science and Control, Hungarian Research Network, Kende u. 13–17, Budapest, 1111 Hungary; 2 Research Center of Vehicle Industry (SZE-JKK), Széchenyi István University, Győr, 9026 Hungary

**Keywords:** Autonomous vehicle, Reinforcement learning, Observer design, Vehicle dynamics and control, Engineering, Mathematics and computing

## Abstract

This paper introduces a novel method for the enhancement of automated vehicle safety and efficiency during critical manoeuvres. The fundamental of the presented method is the observer design architecture, in which lateral dynamic states of the vehicle are evaluated. The novel observer consists of both model-based and machine-learning-based methods to ensure the selected design performances, such as efficient trajectory tracking and safety evaluation of the autonomous vehicle. In contrast to the already introduced and applied stability index-based methods, the proposed safety evaluation process is able detect stability loss and performance degradation of the autonomous vehicle. In the proposed observer-based safety evaluation method, stability and performance loss detection is based on the comparison of model-based and learning-based state observation. The main novelty of the paper is the design of the reinforcement learning (RL) based observer in a guaranteed structure that results in small observation error even under nonlinear vehicle dynamics. Furthermore, a lateral safety index is defined based on the value of the improvement vector representing the addition to the model-based estimation. By this means, with the proposed safety evaluation method both safety and performance loss hazards can be identified simultaneously.

## Introduction

Recent autonomous vehicle developments and research highlighted the importance of high-performance and stable controllers to deal with several re-al-life traffic scenarios. A state estimation based on Kalman filtering combined with cooperative active steering and yaw-moment control has been introduced in^1^ to guarantee lateral stability of autonomous vehicles. For four-wheel independent driven autonomous vehicles, a high-level lateral stability path-tracking model predictive controller (MPC) has been introduced in^2^, where a low-level allocation method generated the desired amount of steering and yaw-moment input. A collision avoidance method for autonomous vehicles containing trajectory tracking, adaptive cruise control (ACC) and active obstacle avoidance has been introduced in^3^, where MPC and non-linear MPC (NMPC) methods have been utilized. Automated vehicles must deal with various tasks to substitute conventional driver intervention, thus sensing, perception and trajectory planning functionalities must be designed, as comparatively reviewed in^4^. A review on deep learning sensor fusion methods has also been performed in^5^, focusing on autonomous vehicle perception and localization issues. An offline trajectory optimization and an NMPC tracking controller has been introduced in^6^, showing effectiveness in a race-line high-speed scenario. As safety is key element of autonomous vehicles, the set of actions and corresponding control inputs must be determined from a safety aspect^7^.

Safety evaluation can be a challenging task however, despite having fundamental vehicle dynamics laws describing stability, as detailed in^8^. Stability and controllability analysis has been carried out in^9^ using a multi-body system dynamic model, while conservative vehicle lateral stability regions have been identified for control applications in^10^.

Firstly, unstable vehicle movements are difficult to identify purely based on vehicle states, as velocity may decrease during several type of unstable motions, which can have a stabilizing effect, as suggested by the well-known Pacejka magic formula tire model in^11^ and the stability study for the nonlinear tire region carried out in^12^. Thus, locally unstable motions may be evaluated as stable. Secondly, even stable vehicle states can result in hazardous scenarios if performance loss is present, thus incorporating tire dynamic characteristics in the control design is essential, see^13^. Moreover, vehicle stability analysis based on the behaviour of the zero dynamics has been derived in^14^, by which the start of instability can be identified. For instance, during high-speed trajectory tracking, differentiating unstable movements and low performance can be challenging^15^. Hence, there is a need to design an evaluation method, by which both types of safety critical problems can be detected. Learning-based solutions are recently applied in data driven modelling and control for highly nonlinear problems^16^.

An adaptive neural network control system for a quarter car electrohydraulic active suspension system dealing with dynamic nonlinearities and uncertainties has been proposed in^17^, showing better performance than conventional proportional–integral–differential (PID) controllers. A novel method combining robust H ∞ control with reinforcement learning using the Proximal Policy Optimization algorithm has been pro-posed in^18^ for autonomous vehicle trajectory tracking task, showing robust lateral and longitudinal tracking with enhanced performances. A survey of reinforcement learning-based techniques for autonomous vehicle trajectory prediction has been introduced in^19^, highlighting their respective strengths and limitations. A Deep Reinforcement Learning (DRL) technique using Deep-Q Networks has been designed in^20^ to control autonomous vehicles in a more complex scenario with other road participants, with the proposed method validated in CARLA simulator.

Several safety evaluation methods connected to lateral dynamics have been introduced already. For example, an integrated vehicle control method has been introduced in^21^, where the active front steering and dynamic stability control systems cooperate to achieve stability of the vehicle during extreme conditions. An observer based and a reinforcement learning based method is combined in^22^, to evaluate safety of the vehicle motion. Unstable movements can also be detected using stability index founded on online evaluation of side-slip^23^, how-ever, performance degradation cannot be detected this way. Offline computation of safety sets with nonlinear^24^ or data-driven^25^ methods has several advantages, however, its relevance during promptly varying dynamical conditions is limited. Furthermore, a method founded on fixed point transformation has been introduced^26^, which eliminates the need for Lyapunov-function-based stability analysis. A comprehensive survey on lateral stability criteria has been presented in^27^, where both linear and nonlinear methods have been listed.

Present paper introduces a novel observer-based safety evaluation method, in which stability and performance loss detection is based on the comparison of model and learning based state observation. The structure of the novel observer design technique has been presented in^28^. As it has been described, for the observation a bounded ∆ vector is given, which performs innovation by the learning-based observation for the robust H∞-based observer. The main novelty of the present paper is the reinforcement learning (RL) based observer design in a guaranteed structure, which results in a small amount of observer error even under nonlinear vehicle dynamics. Furthermore, lateral safety index ϵ is defined founded on the value of ∆. By this means, with the proposed safety evaluation method both safety and performance loss hazards can be identified simultaneously. With the proposed RL based observer design method, the closed-loop performance of automotive controllers can be enhanced.

The paper is organized as follows: First, the architecture of the proposed augmented RL-based observer design is described, along with the safety evaluation method and the operation of these functions in the closed-loop control process. Here, the mathematical formulation of the observer and the improvement gain given by the RL-based observer is also detailed, along with the description of the safety evaluation method responsible for detecting critical situations. Next, simulation examples showing the operation of the proposed augmented RL-based observer and the safety evaluation method are presented, illustrating the effectiveness of the method. Then, training of the RL-based observer using data collected by an autonomous test vehicle at the ZalaZone test track is described. Based on the real-life measurements, the operation of the proposed learning aided observer and safety evaluation method is validated. Finally, concluding remarks and future research challenges in advancing the method for closed-loop control design are given.

## Structure of the state observer for safety evaluation

**Fig. 1:**
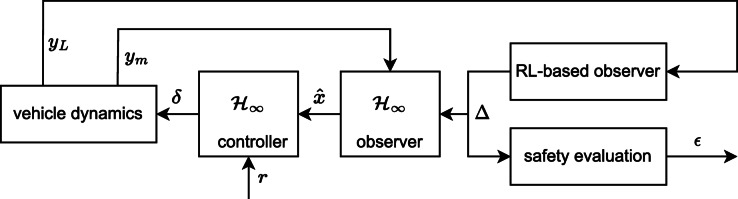
Illustration on the vehicle control architecture within the proposed augmented RL-based observer.

The lateral vehicle safety evaluation is founded on the observation of the vehicle states, as depicted in Fig. 1. Note, that during the observer design the vehicle lateral dynamics are given by the well-known bicycle model equations detailed in^8^:

1 $$\:\left\{\begin{array}{c}J\ddot{\psi\:}={c}_{1}{l}_{f}\left(\delta\:-\beta\:-\dot{\psi\:}{l}_{1}/v\right)-{c}_{2}{l}_{r}\left(-\beta\:+\dot{\psi\:}{l}_{2}/v\right)\\\:mv(\dot{\psi\:}+\dot{\beta\:})={c}_{f}\left(\delta\:-\beta\:-\dot{\psi\:}{l}_{f}/v\right)+{c}_{r}\left(-\beta\:+\dot{\psi\:}{l}_{r}/v\right)\\\:{\ddot{y}}_{v}=v(\dot{\psi\:}+\dot{\beta\:})\end{array}\right.$$

where *β* and *ψ* represents the side-slip and yaw angle of the vehicle, *v* is the velocity, $\:{\ddot{y}}_{v}\:$is the lateral acceleration, *J* is the yaw inertia, *m* is the vehicle mass, *l*_*f*_ and *l*_*r*_ are the distances of the front and rear axles from the vehicle centre of gravity, *c*_*f*_ and *c*_*r*_ are the front and rear tire cornering stiffnesses.

The vehicle states $\:\widehat{x}$ to be observed contains the yaw-rate, lateral velocity and lateral position error from the designed path. The autonomous control architecture contains several elements, having different purposes in the control loop as follows:

- The proposed control scheme contains an RL-based observer with the aim of reflecting nonlinear dynamics of the vehicle. The observer is designed by evaluating episodes using Deep Deterministic Policy Gradient (DDPG) method, see^29^. These episodes consist of various vehicle maneuvers with different longitudinal velocities. The input vector of the observer *y*_*L*_ consists of the front wheel steering angle δ and the measured lateral error *e*_*y, m*_ from the designed trajectory. For both input signals the actual and past values of δ, *e*_*y, m*_ is examined, i.e., the last two values of δ and the last six values of *e*_*y, m*_. The output of the RL-based observer block is noted with ∆ innovation vector for the H ∞ observer.
- The aim of the H ∞ observer is to ensure constrained observation error, even if the RL-observer performance degrades during the training process. Note that the model-based observer is designed using minimal number of measured signals, thus its complexity is limited. Hence, *y*_*m*_ consists of solely the actual lateral error of the vehicle *e*_*y*_. The robust quality of the observer is gained by ∆, which serves as an extension for the computation of the state $\:\widehat{x}$. Note, that during the observer design ∆ is considered as a bounded disturbance.
- Based on the reference trajectory and the output of the observer $\:\widehat{x}$, a steering angle δ is computed for the autonomous vehicle by the H ∞ controller. The robust control design is based on a weighting strategy, in which weighting functions are designed to reflect trajectory tracking and steering angle limitation requirements, see^30^. Note, that the subject of present paper is the design of a learning-aided state observer and a safety evaluation method, hence the robust controller design is not considered here. In the classical linear control theory approach, observer *L* and controller *K* are designed within the same procedure, see^31^. As the well-known separation principle for linear systems are not applicable for H ∞ controller design, a coupled LMI approach should be used to guarantee robustness, however, this might result in a more conservative solution, see^32^. During the design, it is necessary to achieve that the observer poles lie to the left of the controller poles in the complex plane, i.e. the observer convergence rate must be faster than that of the controller.

The aim of the H ∞ observer design is to compute a vector *L* to minimize observation error, hence, to minimize the difference between *x* and $\:\widehat{x\:}\:$in quadratic manner. The observer design is found on the solution of the following algebraic Riccati inequalities:

2 $$\:AY+Y{A}^{T}+Y\left({\gamma\:}^{-2}{C}_{1}^{T}{Q}^{-1}{C}_{1}-\frac{1}{r}{C}_{2}^{T}{C}_{2}\right)Y+{B}_{1}{B}_{1}^{T}<0\:\:$$

$$\:Y>0,$$.

where *A*,* B1*,*C1*,*C2* are the system matrices, *Y* is a symmetric matrix, *Q*,* r* represents tuning parameters^28^, γ > 0 scalar stands for the upper bound of the H ∞ norm of the transfer function from *w* to observation performance. The aim of the observer design is the minimization of γ < 1, to ensure robustness against the disturbances. The result of the minimization is *Y*, from which *L* is computed as follows:

3 $$\:L=\frac{1}{r}{C}_{1}Y$$

The calculation of *L* results in an optimization where γ must be minimized while Eq. (2) must be feasible. The improvement *∆* gained by RL-based observer in the calculation of the state estimation is given as follows:

4 $$\:\dot{\widehat{x}}=A\left(\hat{x}+\varDelta\:\right)+{B}_{2}u+L\left({y}_{m}-{C}_{2}\hat{x}\right)$$

Hence, ∆ is considered as an addition to the estimation of the H ∞ observer derived from $\:\widehat{x}$_L_, in order to minimize the difference between $\:\widehat{x}$_L_ and $\:\widehat{x\:}$. However, this difference is constrained by predefined bounds as follows:

5 $$\:{\varDelta\:}_{i}={{max}\left\{{min}\left({\hat{x}}_{i}-{\hat{x}}_{L,i};{\varDelta\:}_{max,i}\right);{\varDelta\:}_{min,i}\right\}}_{\forall\:i\in\:n}$$

where max, min functionals stand for the selection of the bigger or smaller values.

Note, that ∆ not only improves the observation procedure, but also serves for the safety evaluation of the vehicle. Elements of ∆ vector contain the difference between the linear and the learning-based observation of that given vehicle state. Moreover, as for the learning-based observer training in the nonlinear operation region is also performed, the increment of ∆_i_ stands for increasing distance of the vehicle states from the linear region of operation. Hence, this comparison serves as the foundation of the safety evaluation process.

**Fig. 2:**
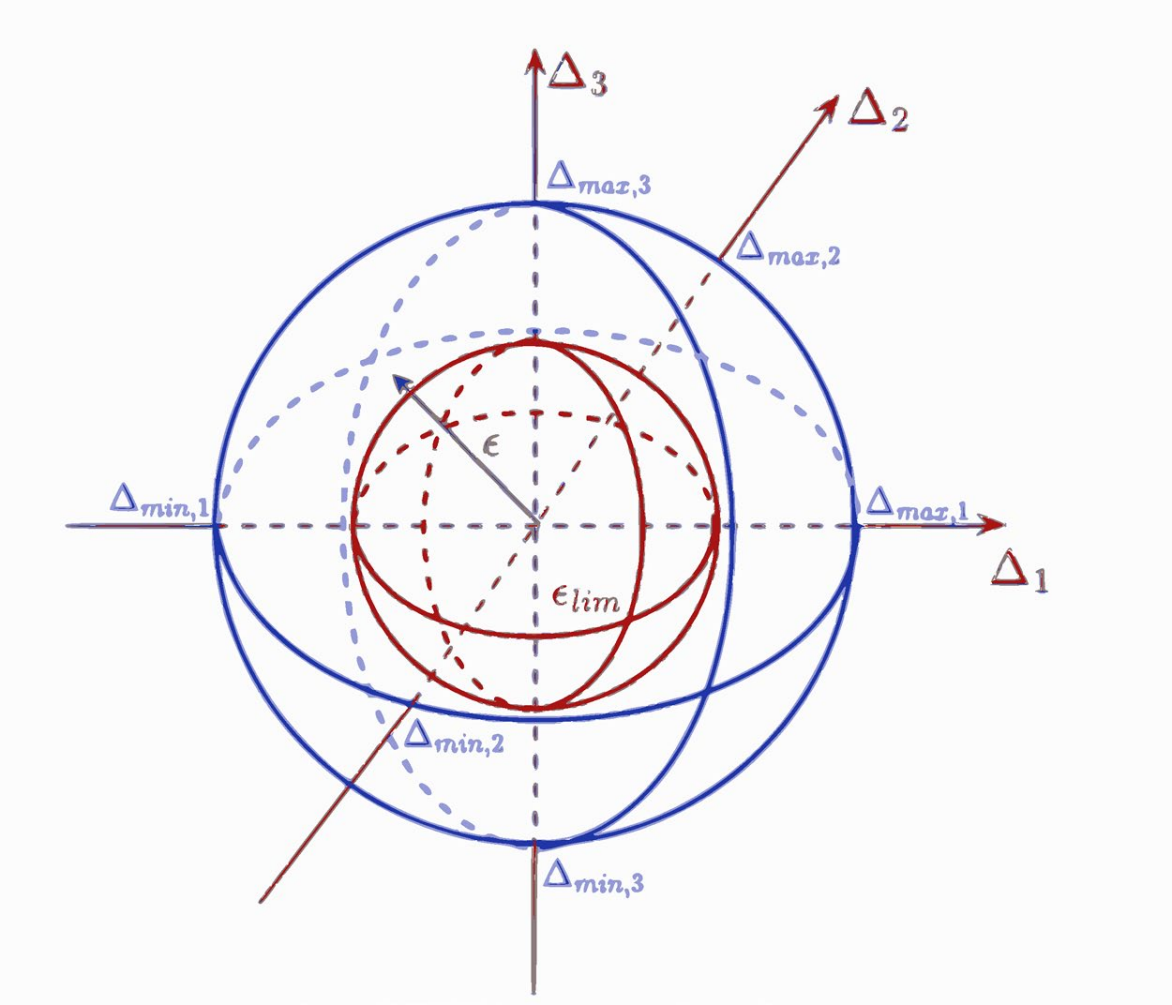
Relationships between ∆_i,ϵ_ and ϵ_lim_.

During the operation of the vehicle control system, value of ϵ = ||∆|| is calculated continuously. Given ∆ is a bounded vector, ϵ has also an upper limit. In case of ϵ > ϵ_lim_, where ϵ_lim_ parameter is close to the upper limit, then a safety critical vehicle motion is detected, i.e. the vehicle is in the nonlinear region. The illustration on the comparison process is depicted in Fig. 2.

If ∆_min, i_=∆_max, i_,∀i, then the bound on ∆ is a sphere, else it has an irregular sphere form, given as a blue shape in Fig. 2. The chosen ϵ_lim_ stands for a sphere, which is pictured with red. The calculated ϵ poses a vector, which can be given in the space. In case the endpoint of this vector ϵ is outside of the inner sphere, then the vehicle is in a safety critical situation.

Note, that detecting safety critical situations enhances the robustness of the autonomous vehicle trajectory control procedure, which is the aim of the presented method. These safety critical situations can occur during driving scenarios where road adhesion vary abruptly, sudden changes occur in vehicle dynamics due to loss of performance such as punctures, or changes in weather conditions affecting the behavior of the autonomous vehicle.

### Simulation examples

First, the presented method is demonstrated through a simulation example. Here, during the training process the test vehicle drove along a curved road with varying speed. The reward function has been given in the manner that estimation errors of the complete observer and of the RL-based observer are present as follows:

6 $$\:r\left(k\right)=-\sum\:_{i=1}^{3}\left(\left|{x}_{i}\left(k\right)-{\hat{x}}_{i}\left(k\right)\right|+\left|{x}_{i}\left(k\right)-{\hat{x}}_{L,i}\left(k\right)\right|\right)$$

**Fig. 3:**
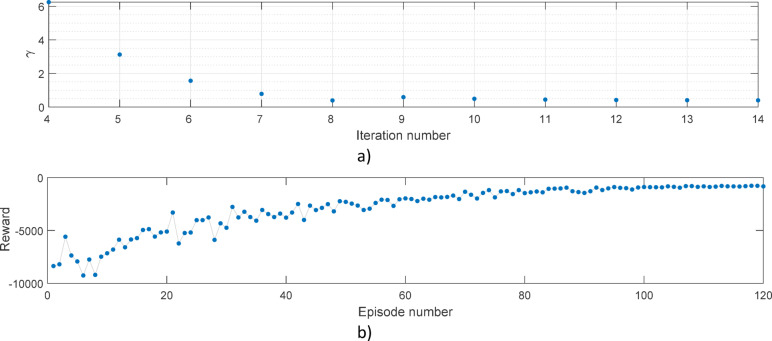
Example of the design procedure. Convergence of (**a**) γ value; (**b**) Cumulative reward.

Note, Learning-aided observer design for improving autonomous vehicle safety that reward *r(k)* is summed in each episode resulting in the cumulative reward. The aim of the training procedure is to maximize the cumulative reward. The results of the H ∞ observer design and the training procedure are illustrated in Fig. 3.

Figure 3a) demonstrates the minimization process on *γ*, which is connected to the performance of the model-based H ∞ observer controller. Note, that *γ < 1* final value has been achieved in the iteration process, thus robustness and feasibility are both guaranteed, as the aim of the observer design is the minimization of *γ*, which describes the upper bound of the H ∞ norm of the transfer function from the disturbance to observation performance. Figure 3b) illustrates the convergence of the cumulative reward computed based on the equation detailed in Eq. 5. As depicted, after 120 episodes a big cumulative reward has been achieved.

**Fig. 4:**
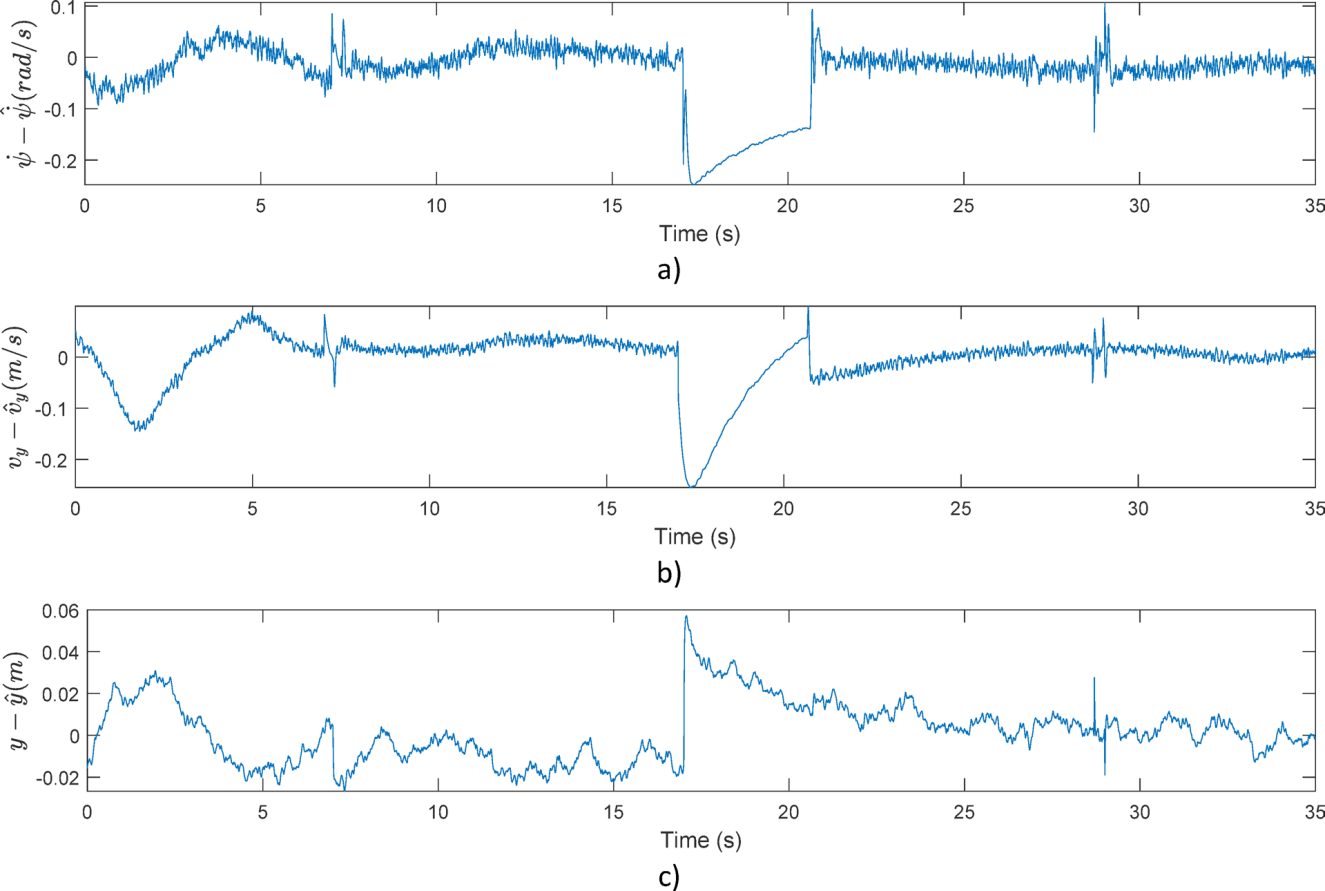
Example observation errors: (**a**) Yaw-rate; (**b**) Lateral velocity; (**c**) Lateral position.

Figure 4 shows a simulation on the performance of the observer. Note, that for every state (yaw-rate, lateral velocity and lateral position) the observation errors are generally kept small, however, during the start of the simulation an increased error is present due to the initial condition errors. Also, between 17–21 s an increased error is achieved by the abrupt lateral movement of the vehicle. This results in a safety-critical situation, which is detected by the proposed method. Note, that with the given method, not only stability loss, but also performance loss (at section between 17–21 s due to steering saturation) can be detected.

**Fig. 5:**
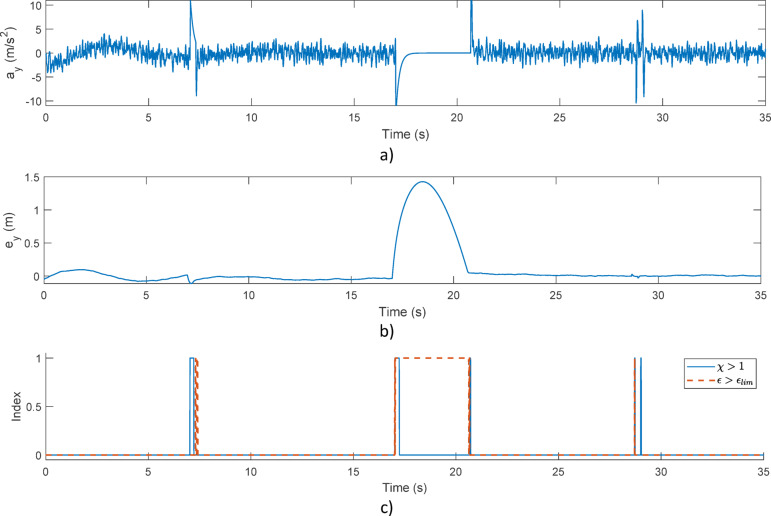
Effectiveness of the proposed method: (**a**) Lateral acceleration; (**b**) Lateral tracking error; (**c**) *χ* and $\epsilon$ indexes.

The simulated safety-critical situation is detected by the proposed evaluation method as shown in Fig. 5. Note, that lateral error of the simulated vehicle has increased during the safety critical motion, as depicted in Fig. 5b). As a result, the safety critical motion from 17 s has been continuously detected, see Fig. 5c). Hence, the presented procedure along with the stability index can be used effectively in the safety evaluation of the vehicle.

### Real-life demonstration of the safety evaluation method

**Fig. 6:**
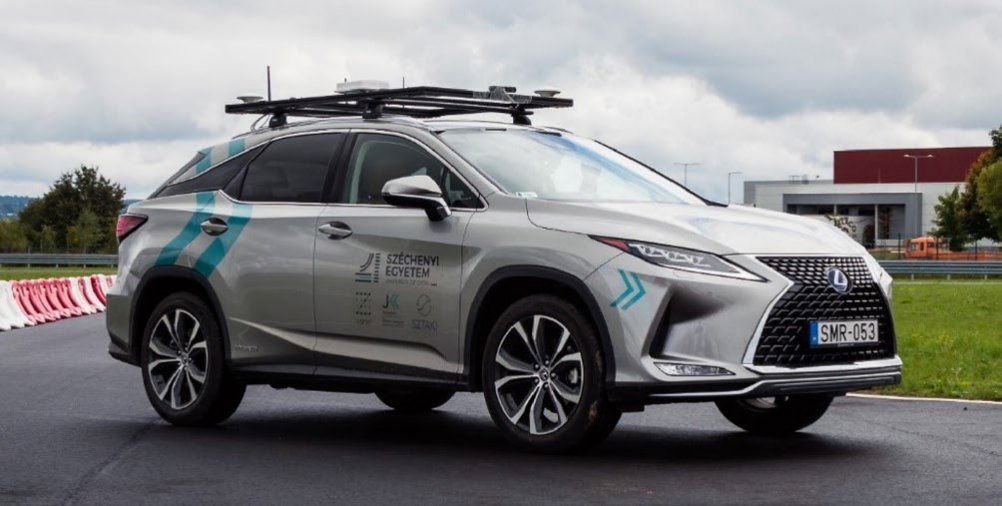
Autonomous test vehicle.

First, training of the RL-based observer has been evaluated based on real-life data collected by the autonomous test vehicle on the ZalaZone test track, see Fig. 6. Data collection was evaluated with a LEXUS RX450h test vehicle equipped with autonomous driving features.

Hence, the implementation is based on a linear single-track (bicycle) lateral vehicle model described in Eq. (1) of the test vehicle with assuming linear tire cornering stiffness. The parameters of the test vehicle used for the observer design is listed in Table 1.

**Table 1:** Autonomous vehicle parameters.

Parameter	Value	Unit
Vehicle mass (*m*)	2200	*kg*
Yaw moment of inertia (*J*)	4000	*kgm* ^2^
Distance from C.G to front axle (*l*_1_)	1.1	*m*
Distance from C.G to rear axle (*l*_2_)	1.7	*m*
Cornering stiffness front (*c*_1_)	132.92	*kN/rad*
Cornering stiffness rear (*c*_2_)	191.34	*kN/rad*

**Fig. 7:**
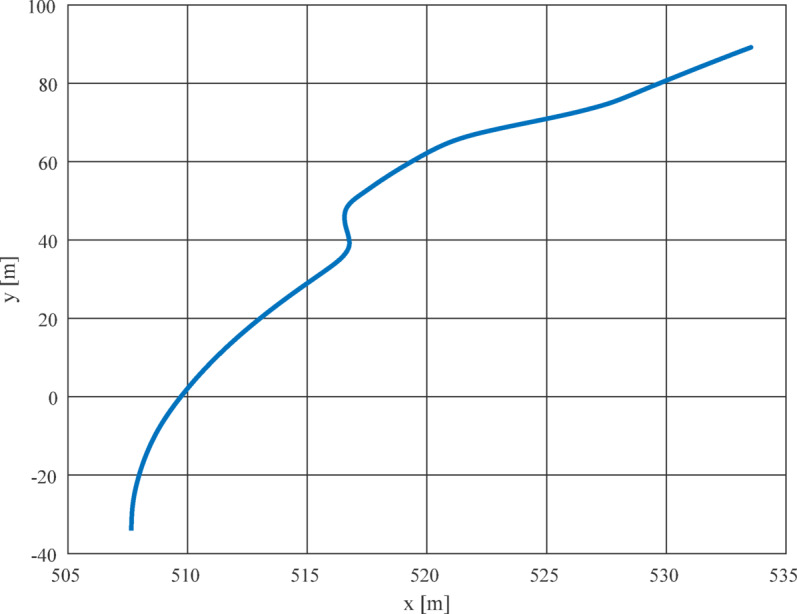
Moose test track.

The measurements have been collected up on the Dynamic Platform section of the test track^33^, where a standardized Moose test has been reproduced with a sudden lane change, as shown in Fig. 7.

During the tests, the vehicle position, velocity, lateral acceleration, yaw-rate, steering angle and lateral velocity have been measured by sensors (GPS, IMU, etc.), while side slip angle and lateral error have been obtained through additional calculations. In the given scenario, the available data types were predefined, necessitating the selection of the optimal combination and the determination of the appropriate number of retrospections for each type, to achieve the best possible results with the agent. Throughout this process, all other variables are kept constant, focusing solely on varying the observation data. The initial focus of the training sessions is to minimize the learning state error.

**Fig. 8:**
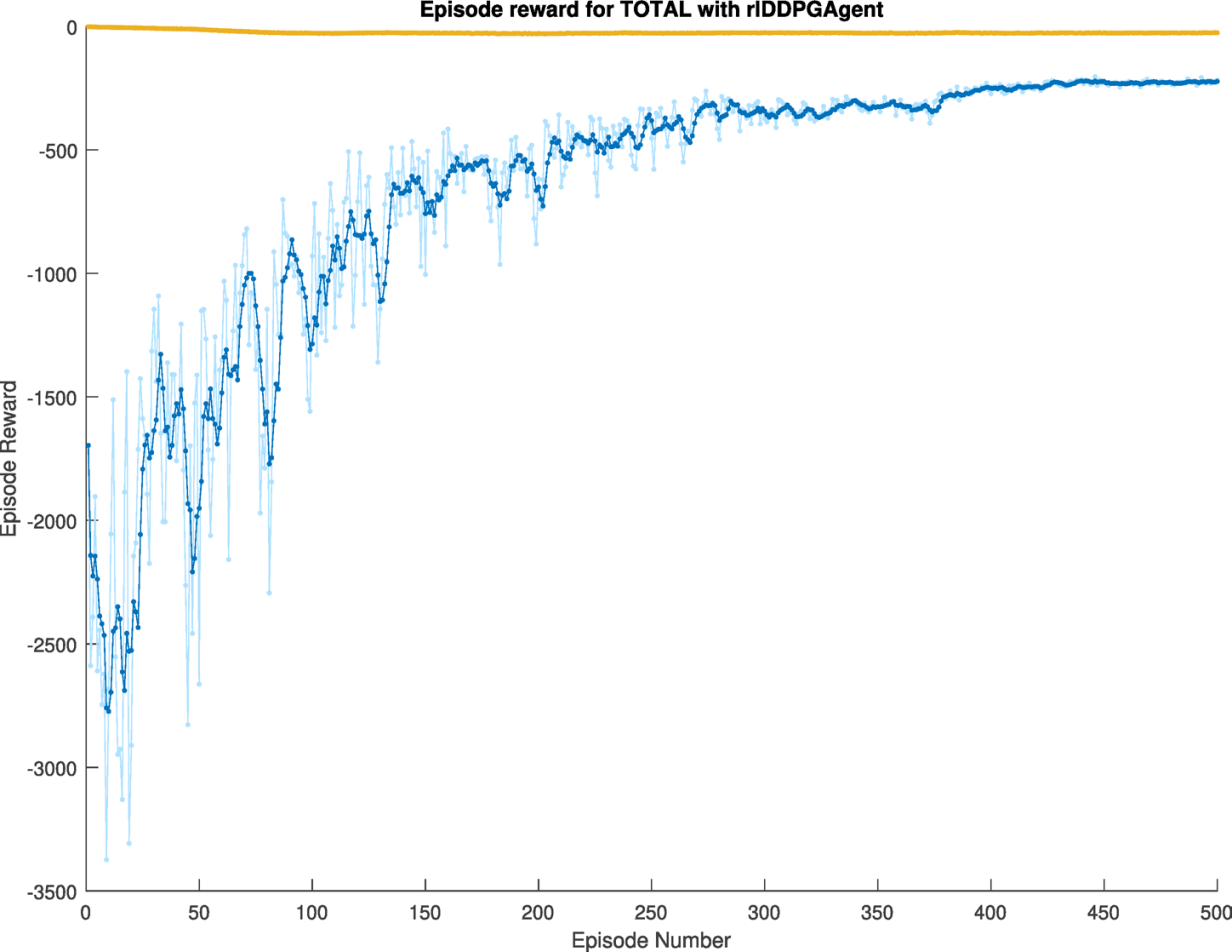
First case learning reward.

The reward of the learning agent is calculated by subtracting the estimated state values, which emerge from the learning process, from the actual state values, and then summing these differences. The objective is to ensure that the training progressively converges in the direction of zero, indicating optimal performance. In the first case, the training utilized the vehicle’s lateral error and steering values, with a retrospective view of five instances for the former and three for the latter. The training ran through 500 episodes and achieved an average reward of −222.75 in the first case, see Fig. 8.

In the second case, all measured values were included in the training to achieve better performance for the observer, thus 15 observations consist in the system (lateral error with 4 delay blocks; steering with 3 delay blocks; velocity with 2 delay blocks; lateral acceleration with 2 delay block). Parameters of the reinforcement learning training algorithm are depicted in Table 2.

**Table 2:** Reinforcement learning parameters.

Parameter	Value
RL algorithm	Deep Deterministic Policy Gradient (DDPG)
Observation dimension	15
Action dimension	3
Actor network	FC(48) – ReLU – FC(48) – ReLU – FC(48) – ReLU – FC(3) – tanh – scaling
Critic network	State: FC(48)–ReLU–FC(48); Action: FC(48); merged → FC(48)–ReLU–FC(1)
Actor learning rate	1.00E-04
Critic learning rate	1.00E-03
Discount factor (gamma)	0.99
Target smoothing factor (tau)	1.00E-03
Replay buffer size	1.00E + 06
Mini-batch size	64
Exploration noise variance	0.6
Exploration noise variance decay	1.00E-05

**Fig. 9:**
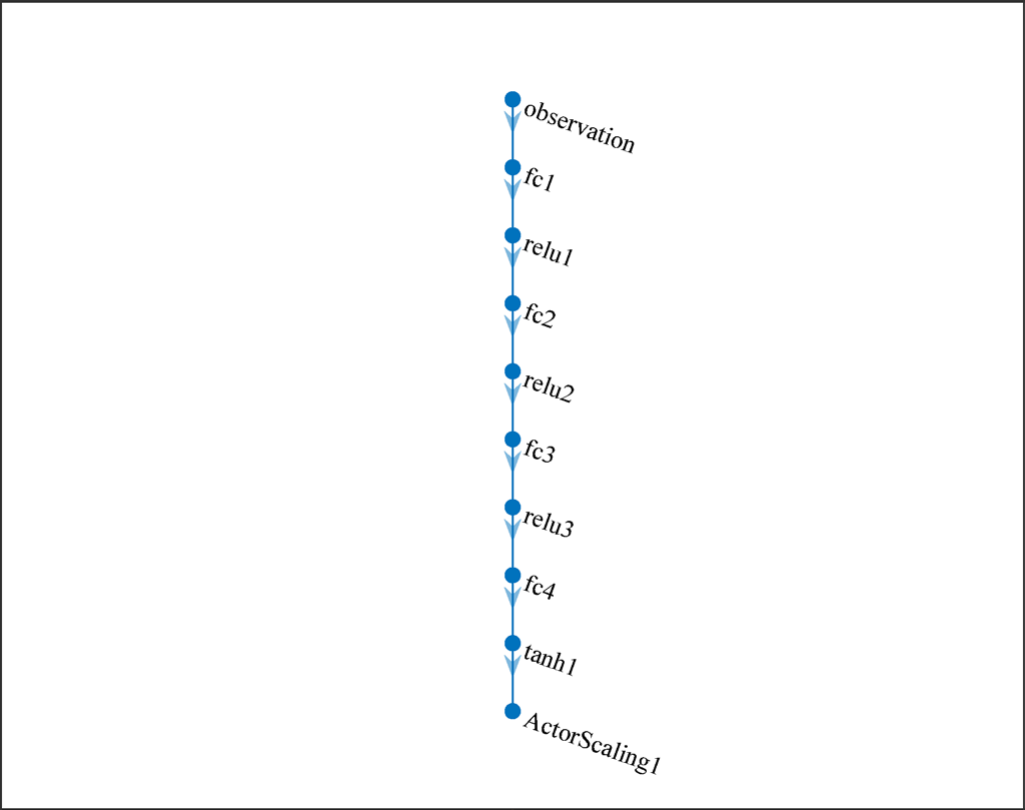
Architecture of the actor-critic network.

The training loop of the previously defined RL is illustrated in Fig. 9. As the agent selects its actions based on the current policy and its interactions with the environment, these actions generate new states and generate a reward which are fed back to the agent. Founded on this feedback, the policy of the agent is updated to improve the decision-making process, and this iterative cycle helps the agent to maximize the cumulative reward.

**Fig. 10:**
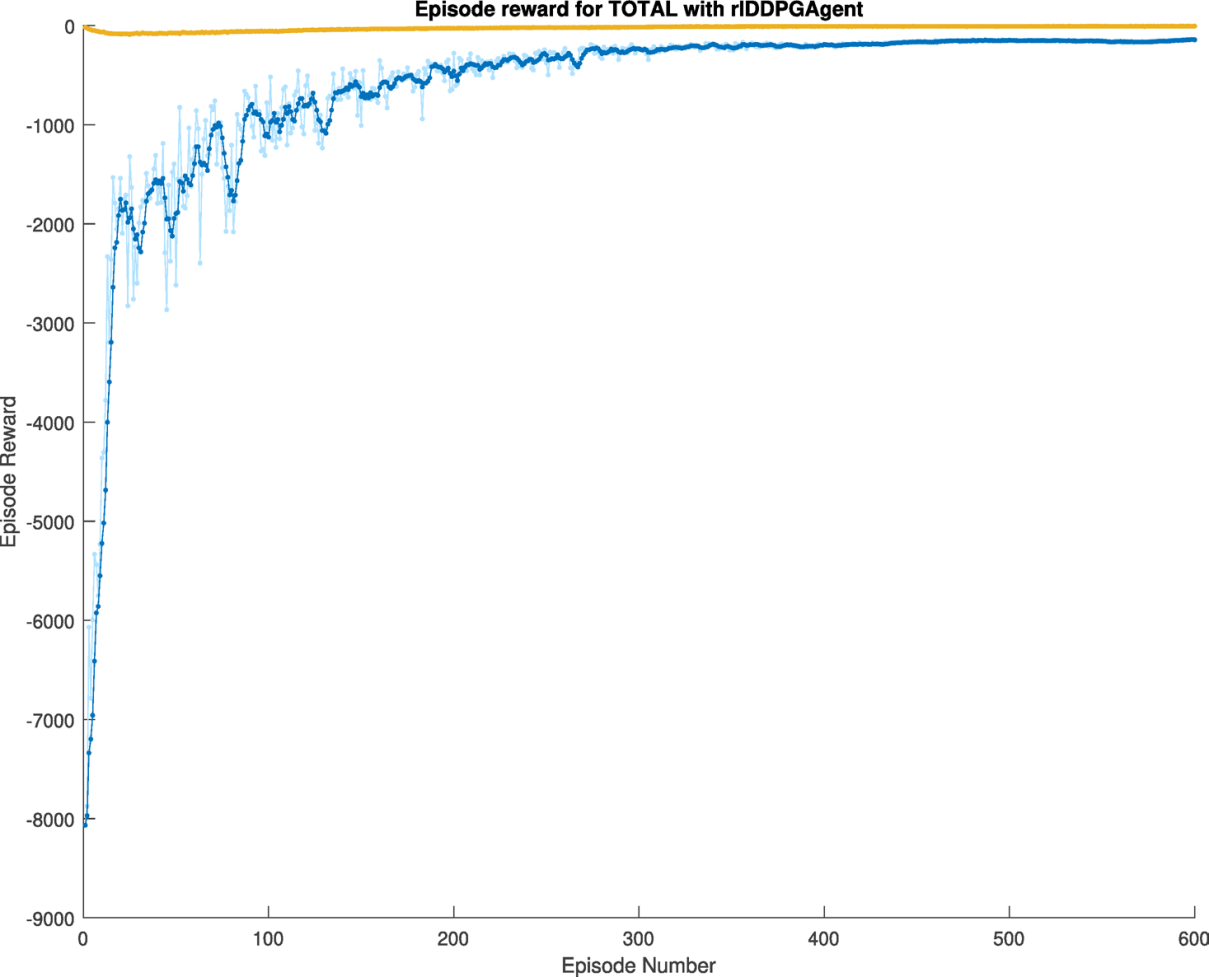
Second case learning reward.

The reward curve achieved in this session shown in Fig. 10 consistently converged towards the direction of zero throughout the training, with an average reward value of −151.80 and episode reward of −142.36, showing significantly better performance than in the first case.

**Fig. 11:**
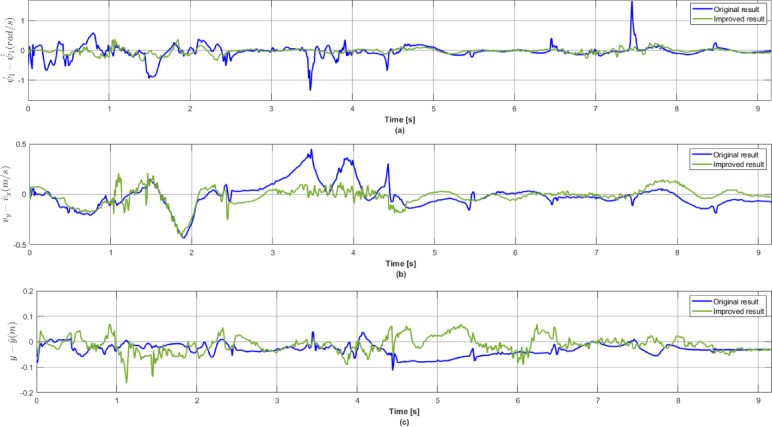
Comparison of the original and the improved observation space in terms of state error in one simulation case: (**a**) Yaw-rate; (**b**) Lateral velocity; (**c**) Lateral position.

Figure 11 illustrates the differences in state estimations by the agent trained with the original observation versus the results achieved with the improved structure. With the improved version, the system achieved significantly better state estimation results. Figure 11a) demonstrates that yaw-rate estimation error with the improved system is significantly smaller, with the spikes at 3.5 and 7.5 s disappearing successfully. Moreover, lateral velocity error depicted in Fig. 11b) and lateral position error shown in Fig. 11c) also improved, especially during higher lateral accelerations.

The better estimation results of the improved structure are also expressed with statistical measures listed in Table 3, showing the Mean Absolute Error (MAE), Standard Deviation (STD) and Root Mean Square Error (RMSE). It is well demonstrated that all the state estimations have improved significantly, with greatly reduced estimation error for all the statistical measures.

**Table 3:** Reinforcement learning parameters.

		MAE	STD	RMSE
Original	yaw-rate	0.5141	0.6281	0.6495
	lateral acceleration	0.1957	0.2642	0.2768
	lateral error	0.3015	0.4453	0.4779
Improved	yaw-rate	0.2110	0.2566	0.2703
	lateral acceleration	0.1003	0.1370	0.1390
	lateral error	0.0694	0.0784	0.0925

**Fig. 12:**
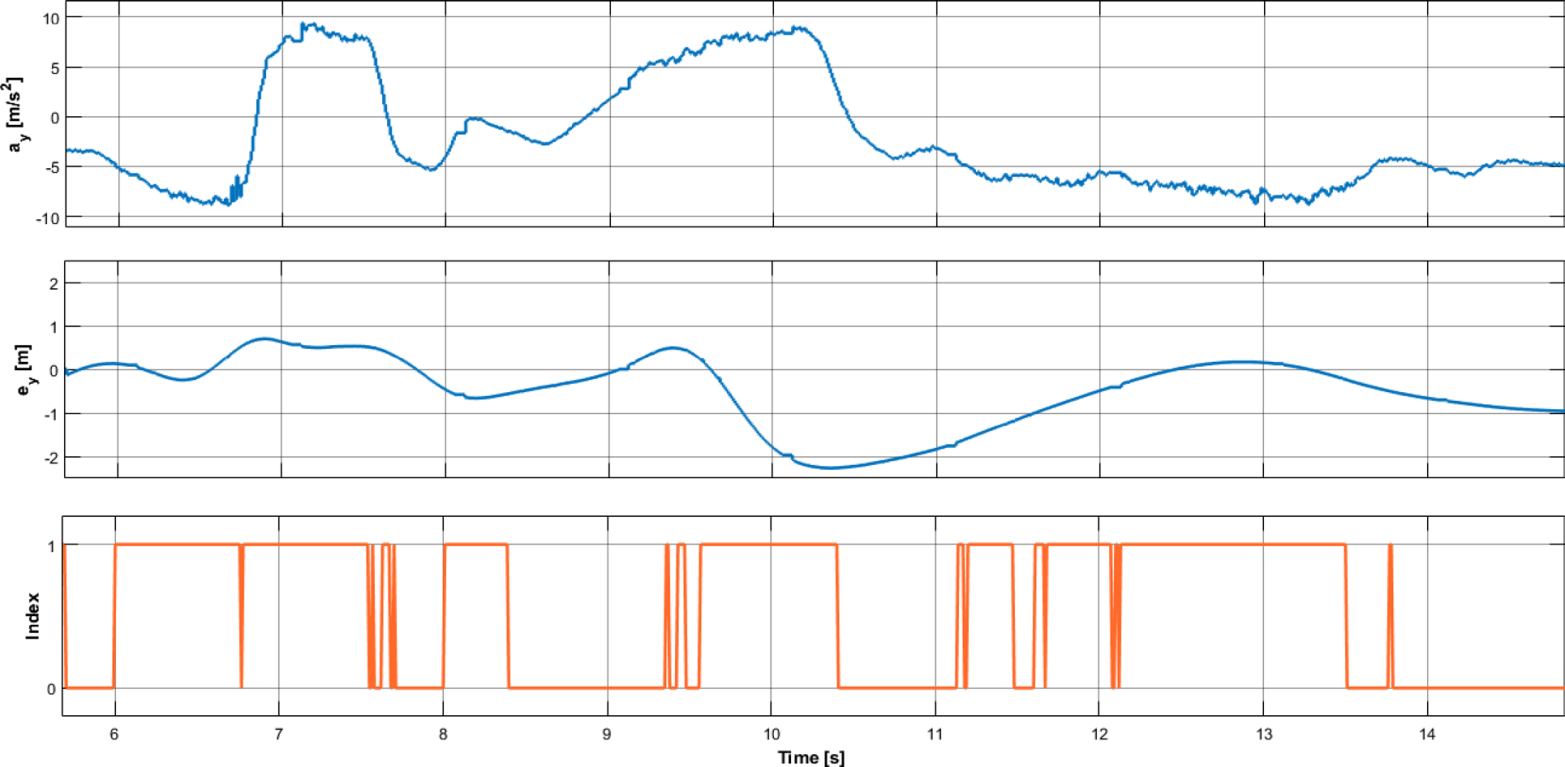
Effectiveness of the proposed method: (**a**) Lateral acceleration; (**b**) Lateral tracking error; (**c**) safety index.

Figure 12 depicts the test results obtained with the improved agent. Figure 12a) presents the vehicle’s lateral acceleration, while Fig. 12b) shows the vehicle’s lateral error. Figure 12c) depicts the safety-critical index, that is, whether the algorithm classifies the given situation as dangerous.

Similarly to the former simulation scenario, it can be seen here as well that the proposed method can indicate hazardous situations over their entire duration.

## Conclusion

The paper presented a novel safety evaluation method in order to detect safety critical events, such as lateral stability loss and degradation in trajectory tracking performance. The main advantage of the proposed procedure is that both safety hazards can be detected at the same time. The method is founded on the comparison of a robust model-based and a reinforcement-learning-based observer. As the critical situation detection is independent from the internal structure of the RL-based observer, different types of learning-based agents can be applied. It has been shown based on test measurements, that the design of the reinforcement learning agent has big effect on the observer performance. Moreover, the proposed safety evaluation method creates an opportunity to identify any hazardous vehicle movement. As present paper does not deal with stabilization of the vehicle in these critical vehicle states, in future work the result of the presented safety evaluation can also be used as feedback for the vehicle dynamic controller to enhance its performance. In case of closing the loop using a robust controller, the design process should also consider a coupled LMI approach to ensure robustness of the joint observer-controller system, while convergence rate of the observer should also be considered by carefully selecting faster poles than that of the controller.

## Data Availability

All data generated or analysed during this study is available from the corresponding author on reasonable request.

## References

[1] Liu, J., Liu, H., Wang, J. & Gu, H. Coordinated lateral stability control of autonomous vehicles based on state Estimation and path tracking. *Machines* **11**, 328. 10.3390/machines11030328 (2023).

[2] Yu, Z., Zhao, R. & Yuan, T. Lateral-stability-oriented path-tracking control design for four-wheel independent drive autonomous vehicles with tire dynamic characteristics under extreme conditions. *World Electr. Veh. J.* **15**, 465. 10.3390/wevj15100465 (2024).

[3] Cai, J., Yang, S. & Guang, H. A review on scenario generation for testing autonomous vehicles. *Proc. Intell. Veh. Symp. (IV)*, Jeju Island, Korea, 3371–3376 (2024). 10.3390/s24165211

[4] Saqib, N., Yousuf, M. M. & Rashid, M. Design and implementation issues in autonomous vehicles—A comparative review. *Proc. 2nd Int. Conf. Comput., Autom. Knowl. Manag. (ICCAKM)*, Dubai, UAE, 157–162 (2021). 10.1109/ICCAKM50778.2021.9357706

[5] Fayyad, J., Jaradat, M. A., Gruyer, D. & Najjaran, H. Deep learning sensor fusion for autonomous vehicle perception and localization: A review. *Sensors* **20**, 4220. 10.3390/s20154220 (2020). 32751275 10.3390/s20154220 PMC7436174

[6] Li, J. T., Chen, C. K. & Ren, H. Time-optimal trajectory planning and tracking for autonomous vehicles. *Sensors* **24**, 3281. 10.3390/s24113281 (2024). 38894073 10.3390/s24113281 PMC11174940

[7] Dong, D., Ye, H., Luo, W., Wen, J. & Huang, D. Collision avoidance path planning and tracking control for autonomous vehicles based on model predictive control. *Sensors* **24**, 5211. 10.3390/s24165211 (2024). 39204907 10.3390/s24165211 PMC11359412

[8] Rajamani, R. *Vehicle Dynamics and Control* 2nd edn (Springer, 2011).

[9] Hu, L., Fang, S. & Yang, J. Study of the vehicle controllability and stability based on multi-body system dynamics. *Open. Mech. Eng. J.* **8**, 865–871. 10.2174/1874155X01408010865 (2014).

[10] Alves, J., Chinelato, C. & Angelico, B. Vehicle lateral stability regions for control applications. *IEEE Access.* **10**, 1–1. 10.1109/ACCESS.2022.3199752 (2022).

[11] Pacejka, H. B. & Bakker, E. The magic formula tire model. *Veh. Syst. Dyn.* **21** (suppl. 1), 1–18. 10.1080/00423119208969994 (1992).

[12] Liu, L. Vehicle planar motion stability study for tires working in extremely nonlinear regions. *Chin. J. Mech. Eng.* **23**, 185–194. 10.3901/CJME.2010.02.185 (2010).

[13] Koné, T. F., Bonjour, E., Levrat, E., Mayer, F. & Géronimi, S. An approach to guide the search for potentially hazardous scenarios for autonomous vehicle safety validation. *Appl. Sci.* **13**, 6717. 10.3390/app13116717 (2023).

[14] Hu, X. et al. Vehicle stability analysis by zero dynamics to improve control performance. *IEEE Trans. Control Syst. Technol.* **31**, 2365–2379. 10.1109/TCST.2023.3257682 (2023).

[15] Pierini, M. et al. Trajectory tracking for high-performance autonomous vehicles with real-time model predictive control. *Proc. 16th Int. Symp. Adv. Veh. Control (AVEC)*. **Lecture Notes in Mechanical Engineering (Springer)**10.1007/978-3-031-70392-8_2 (2024).

[16] Du, L. et al. A learning-based nonlinear model predictive control approach for autonomous driving. *Proc. 22nd IFAC World Congr*. **2792-2797**10.1016/j.ifacol.2023.10.1388 (2023).

[17] Al Aela, A. M., Kenne, J. P. & Mintsa, H. A. Adaptive neural network and nonlinear electrohydraulic active suspension control system. *J. Vib. Control*. **28**, 243–259. 10.1177/1077546320975979 (2022).

[18] Lelkó, A., Németh, B. & Gáspár, P. Reinforcement learning-based robust vehicle control for autonomous vehicle trajectory tracking. *Eng. Proc.* **79**, 30. 10.3390/engproc2024079030 (2024).

[19] Bharilya, V. & Kumar, N. A survey of the state-of-the-art reinforcement learning-based techniques for autonomous vehicle trajectory prediction. *Proc. Int. Conf. Electr. Electron. Commun. Comput. (ELEXCOM)*. 1–6. 10.1109/ELEXCOM58812.2023.10370504 (2023). Roorkee, India.

[20] Elallid, B. B. et al. A reinforcement learning based approach for controlling autonomous vehicles in complex scenarios. *Proc. Int. Wireless Commun. Mobile Comput. (IWCMC)*, Marrakesh, Morocco, 1358–1364 (2023). 10.1109/IWCMC58020.2023.10182377

[21] He, J., Crolla, D. A., Levesley, M. C. & Manning, W. J. Coordination of active steering, driveline, and braking for integrated vehicle dynamics control. *Proc. Inst. Mech. Eng. D J. Automob Eng.* **220**, 1401–1420. 10.1243/09544070JAUTO265 (2006).

[22] Németh, B., Gáspár, P. & Hrgetić, M. Safety evaluation method for lateral dynamics of automated vehicles. Adv. Dyn. Veh. Roads Tracks III. IAVSD 2023, Lecture Notes in Mechanical Engineering (Springer, 10.1007/978-3-031-66968-2_68 (2024).

[23] Vu, V. T., Sename, O., Dugard, L. & Gáspár, P. Enhancing roll stability of heavy vehicles by LQR active anti-roll bar control using electronic servo-valve hydraulic actuators. *Veh. Syst. Dyn.* **55**, 1–25. 10.1080/00423114.2017.1317822 (2017).

[24] Masouleh, M. I. & Limebeer, D. J. N. Region of attraction analysis for nonlinear vehicle lateral dynamics using SOS programming. *Veh. Syst. Dyn.* **56**, 1118–1138. 10.1080/00423114.2017.1409429 (2018).

[25] Fényes, D., Németh, B. & Gáspár, P. Design of LPV control for autonomous vehicles using the contributions of big data analysis. *Int. J. Control*. **95**, 1–23. 10.1080/00207179.2021.1876922 (2021).

[26] Tar, J. K., Bitó, J. F., Nádai, L. & Tenreiro Machado, J. A. Robust fixed point transformations in adaptive control using local basin of attraction. *Acta Polytech. Hung.* **6**, 21–37 (2009).

[27] Zhewei, Z., Xiaolin, T., Yechen, Q., Yiwei, H. & Ehsan, H. A survey of lateral stability criterion and control application for autonomous vehicles. *IEEE Trans. Intell. Transp. Syst.* **24**, 1–18. 10.1109/TITS.2023.3280200 (2023).

[28] Németh, B., Hegedűs, T. & Gáspár, P. Design framework for achieving guarantees with learning-based observers. *Energies* **14**, 2039. 10.3390/en14082039 (2021).

[29] Ruan, J. et al. The application of machine learning-based energy management strategy in a multi-mode plug-in hybrid electric vehicle, part II: deep deterministic policy gradient algorithm design for electric mode. *Energy* **269**, 126792. 10.1016/j.energy.2023.126792 (2023).

[30] Gáspár, P., Szabó, Z., Németh, B. & Bokor, J. *Robust Control Design for Active Driver Assistance Systems: A Linear-Parameter-Varying Approach* (Springer, 2017).

[31] Gahinet, P. & Apkarian, P. A linear matrix inequality approach to H ∞ control. *Int. J. Robust. Nonlinear Control*. **4**, 421–448. 10.1002/rnc.4590040403 (1994).

[32] Karimi, H. Observer-based mixed H2/H ∞ control design of linear systems with time-varying delays: an LMI approach. *Int. J. Control Autom. Syst.* **6**, 1–14 (2008).

[33] Fehér, Á., Aradi, S., Bécsi, T., Gáspár, P. & Szalay, Z. Proving ground test of a DDPG-based vehicle trajectory planner. *Proc. Eur. Control Conf. (ECC)*, St. Petersburg, Russia, 332–337 (2020). 10.23919/ECC51009.2020.9143675

